# Fermented *Dendrobium officinale* Ameliorates Sleep Deprivation-Induced Depressive-like Behaviors by Attenuating Neuroinflammation and Restoring 5-HT Synthesis via the Gut–Brain Axis

**DOI:** 10.3390/foods15122237

**Published:** 2026-06-21

**Authors:** Youmeng Chen, Xiaojie Zheng, Xin Zhang

**Affiliations:** 1 Department of Food Science and Engineering, Ningbo University, Ningbo 315211, China; 2 Department of Agriculture and Biotechnology, Wenzhou Vocational College of Science and Technology, Wenzhou 325006, China

**Keywords:** *Dendrobium officinale*, fermentation, 5-HT, gut microbiota, depression

## Abstract

Chronic sleep deprivation (SD) disrupts gut–brain axis (GBA) homeostasis and is closely associated with gut microbiota dysbiosis, neuroinflammation, and depression-like behaviors. This study investigated whether fermentation enhances the antidepressant-like effects of *Dendrobium officinale* by comparing fermented *Dendrobium officinale* (FDO) with unfermented *Dendrobium officinale* (DO) in a chronic SD mouse model. FDO significantly ameliorated anxiety and depressive-like behaviors in SD mice. It reshaped gut microbial structures, enriched beneficial bacteria taxa such as *Dubosiella*, *[Eubacterium]_coprostanoligenes_group*, and *Allobaculum*, and increased SCFA levels. FDO also enhanced colonic ZO-1 and Occludin expression and reduced serum levels of LPS and the pro-inflammatory cytokines. At the central nervous system level, FDO inhibited the activation of hippocampal microglia and astrocytes; alleviated neuroinflammation; restored hippocampal TPH2, 5-hydroxytryptamine (5-HT), and 5-HIAA levels; and modulated the 5-HT_1A_/5-HT_2A_ receptor balance. In addition, FDO upregulated BDNF, PSD-95, and SYN expression and reduced corticosterone (CORT) levels. Compared with DO, FDO showed more pronounced regulatory effects. Correlation analysis suggested that 5-HT may link gut microbial metabolites, inflammation, and synaptic plasticity. In summary, these findings support FDO as a potential GBA-targeted functional food for SD-related depressive-like behaviors.

## 1. Introduction

Sleep deprivation (SD) generally refers to the use of artificial interventions to prevent experimental subjects from entering a sleep state, with the aim of investigating the role of sleep in physiological function, neural regulation, and its effects on cognition, emotion, and the immune system [1]. However, in the context of the high-stress and fast-paced nature of modern life, SD is no longer confined to laboratory intervention but has gradually become an important factor affecting physical and mental health in the general population [2]. Numerous studies have shown that SD can induce mood disturbances through multiple pathways, including excitatory synaptic dysfunction, microglial activation, neurotransmitter imbalance, and neurobehavioral abnormalities, all of which are considered important risk factors for the development of depression [3]. At present, conventional antidepressants, such as monoamine oxidase inhibitors and selective serotonin reuptake inhibitors, remain important clinical options for the treatment of depressive disorders. However, adverse effects such as nausea and vomiting, changes in body weight, and disruption of sleep architecture limit their long-term use to some extent [4]. Therefore, there is an urgent need to develop safe and effective intervention strategies for SD-related depressive-like behaviors. Notably, transplantation of the gut microbiota from SD mice into healthy recipients can induce similar depressive-like behaviors in host animals [5], suggesting that gut microbial dysbiosis may represent an initiating event in SD-induced neurobehavioral abnormalities.

Increasing evidence indicates that the microbiota–gut–brain axis, a bidirectional communication network linking the gut microbiota and the central nervous system, is a key pathway regulating host emotion and cognitive function [6]. Clinical studies have shown that increased abundance of opportunistic pathogens and marked depletion of beneficial bacteria are frequently observed in patients with depression and in murine models of depression [7]. Antibiotic treatment can also induce pronounced anxiety-like behaviors in the light-dark box test [8]. In addition, microbial dysbiosis further impairs intestinal mucosal barrier function, facilitating the translocation of lipopolysaccharide (LPS) into the peripheral circulation. The resulting systemic immune response enables peripheral pro-inflammatory cytokines to cross the blood–brain barrier (BBB), thereby triggering central neuroinflammation. This inflammatory response not only directly disrupts synaptic plasticity but also significantly suppresses the expression of brain-derived neurotrophic factor (BDNF) and the synthesis and release of neurotransmitters, including dopamine, 5-HT, and γ-aminobutyric acid [9]. In contrast, SCFAs as core protective mediators of the GBA, play a crucial role in maintaining gut–brain immune and endocrine homeostasis. Butyrate, in particular, can effectively counteract neuroinflammation-driven pathological injury by upregulating BDNF levels and promoting the expression of synaptic plasticity-related proteins, including PSD-95 and synaptophysin (SYN), thereby playing an important role in maintaining emotional homeostasis [10].

Notably, both inflammatory responses and microbial metabolites can influence the central 5-HT system [11]. 5-HT is an important monoaminergic neurotransmitter involved in the regulation of emotion, sleep, and stress responses. Its functional homeostasis depends not only on presynaptic synthesis mediated by tryptophan hydroxylase 2 (TPH_2_), but also on the precise transmission of postsynaptic receptor signaling [12]. Studies have shown that mice deficient in the 5-HT_1A_ receptor exhibit increased depressive-like, fear-related, and freezing behaviors [13], whereas postnatal overexpression of the 5-HT_1A_ receptor in the forebrain can rescue the abnormal behavioral phenotype of knockout mice [14], suggesting an important role of the 5-HT_1A_ receptor in maintaining emotional homeostasis. In addition, dysfunction of the 5-HT_2A_ receptor is closely associated with the pathological processes of multiple neuropsychiatric disorders. Certain 5-HT_2A_ receptor agonists, such as lysergic acid diethylamide (LSD) and psilocybin, can produce rapid antidepressant effects by promoting neuroplasticity [15]. These findings indicate that abnormalities in 5-HT receptor-mediated neurotransmission may be one of the important mechanisms underlying depressive-like behaviors. However, direct use of these agents is often limited by concerns related to psychoactivity, safety, or tolerability, which restricts their broader application. Therefore, modulation of the GBA to improve gut microbial ecology, inflammatory status, 5-HT synthesis, and receptor signaling homeostasis may provide a milder and more sustainable strategy for the intervention of SD-related depressive-like behaviors.

*Dendrobium officinale*, a perennial herbaceous plant of the genus *Dendrobium* in the *Orchidaceae* family, is rich in active constituents such as polysaccharides, amino acids, and alkaloids, and exhibits diverse biological activities, including antioxidant, immunomodulatory, anti-inflammatory, and neuroprotective effects [16,17]. Experimental studies have shown that *Dendrobium officinale* extract can alleviate depressive-like behaviors, potentially through mechanisms involving activation of the noradrenergic and dopaminergic systems, inhibition of oxidative stress, and repair of CORT-induced hippocampal injury [18]. However, some active constituents derived from *Dendrobium officinale* have low direct bioavailability in vivo, and their pharmacological effects are highly dependent on metabolic transformation by the gut microbiota [19]. To overcome this limitation in biotransformation, probiotic fermentation technology has shown substantial advantages. Fermentation not only degrades herbal constituents into small-molecule metabolites that are more readily absorbed but also promotes the synergistic effects between probiotics and substrates, thereby profoundly reshaping the host gut microbiota and amplifying their anti-inflammatory potential [20]. Based on this rationale, the present study used SD mice as a model to systematically evaluate the antidepressant-like efficacy of FDO and DO. Focusing on the gut microbiota, SCFAs, the 5-HT system, peripheral inflammation, glial cells, and synaptic plasticity-related proteins, this study aimed to establish the central role of 5-HT homeostasis in SD pathology and to provide a solid translational basis for FDO as a novel GBA-targeted intervention.

## 2. Materials and Methods

### 2.1. Materials and Reagents

*Dendrobium officinale* stems are provided by Zhejiang Tiefengtang Health Technology Co., Ltd. (Wenzhou, China). *Lactobacillus plantarum* and *Lactobacillus acidophilus*: Henan Provincial Engineering Research Center for Industrial Microbial Strains (Zhengzhou, China). Fluoxetine was purchased from MedChemExpress (South Brunswick, NJ, USA). The experimental mice were obtained from Beijing Vital River Laboratory Animal Technology Co., Ltd. (Beijing, China). Standard animal chow was provided by Ningbo Experimental Animal Center. ELISA kits for interleukin-1β (IL-1β, Cat. No. E-EL-M0037), tumor necrosis factor-α (TNF-α, Cat. No. E-EL-M3063), interleukin-6 (IL-6, Cat. No. E-EL-M0044), 5-HT (Cat. No. E-EL-0033), 5-hydroxyindole-3-acetic acid (5-HIAA, Cat. No. E-EL-0075), and CORT (Cat. No. E-OSEL-M0001) were purchased from Wuhan Elabscience Biotechnology Co., Ltd. (Wuhan, China). ELISA kits for LPS (Cat. No. F2631-B), BDNF (Cat. No. F2204-B), and interleukin-10 (IL-10, Cat. No. F2171-B) were purchased from Shanghai Kexing Biotechnology Co., Ltd. (Shanghai, China).

### 2.2. Preparation and Chemical Characterization of DO and FDO

The *Dendrobium officinale* stems were rinsed, mixed with deionized water at a solid-to-liquid ratio of 1:25 (*w*/*v*), and then the juice was extracted using a cell-breaking machine and filtered through a 0.45 μm cellulose acetate membrane. The resulting filtrate was sterilized in an autoclave at 121 °C for 10 min, and then cooled to room temperature. *Lactobacillus plantarum* and *Lactobacillus acidophilus* were inoculated into MRS broth at a ratio of 1:1.2 (*v*/*v*). They were incubated at 37 °C for 48 h and subcultured once. After activation, the cultures were continuously propagated to the third generation, washed three times with sterile saline, and adjusted to an initial bacterial concentration of 2.6 × 10^8^ CFU/mL. The activated lactic acid bacteria were inoculated into the sterilized Dendrobium officinale juice at an inoculum volume of 5.3% (*v*/*v*). Fermentation was performed at 37.1 °C for 35.6 h, according to the optimized conditions obtained from single-factor experiments and response surface methodology.

To characterize the compositional changes induced by fermentation, total sugar, total acid, total phenolics, and γ-aminobutyric acid (GABA) contents were determined in both DO and FDO samples. Total sugar content was measured using the phenol–sulfuric acid colorimetric method [21]. Total acid content was determined according to GB 12456-2021 and expressed as lactic acid equivalents [22]. Total phenolic content was measured using the Folin–Ciocalteu method [23]. GABA content was determined by high-performance liquid chromatography (HPLC). Briefly, the samples were centrifuged and filtered, followed by pre-column derivatization with o-phthalaldehyde. Separation was performed on a C18 chromatographic column, and detection was carried out using a UV detector. GABA was quantified by comparison with an external standard, and the results were expressed as mg/mL.

### 2.3. Animal Experimental Design

Male specific pathogen-free (SPF) C57BL/6J mice aged 6–8 weeks were provided by Beijing Vital River Laboratory Animal Technology Co., Ltd. (Beijing, China). Their initial body weights ranged from 18 to 20 g at the beginning of the experiment. Male mice were used to minimize potential variability associated with the estrous cycle in female mice and to ensure consistency in behavioral and physiological assessments. The mice were housed in the SPF barrier facility of the Laboratory Animal Center of Ningbo University under controlled conditions of 22 ± 2 °C and 55 ± 5% humidity. After 1 week of acclimatization, the mice were randomly divided into five groups, with 10 mice in each group: the normal control group (NC), sleep deprivation group (SD), sleep deprivation plus fluoxetine group (FLX), sleep deprivation plus *Dendrobium officinale* group (DO), and sleep deprivation plus fermented *Dendrobium officinale* group (FDO). Mice in the NC and SD groups were administered 0.2 mL of normal saline. Mice in the DO and FDO groups were administered 0.2 mL of the corresponding preparation by gavage at a dose of 100 mg/kg body weight (BW) once daily for 5 consecutive weeks. The dose was selected based on previous pharmacological studies showing that *Dendrobium officinale* extract exerts effective immunomodulatory and anti-inflammatory effects in mice at 100 mg/kg [24]. Mice in the FLX group were administered 0.2 mL of fluoxetine at a dose of 20 mg/kg BW once daily for 5 consecutive weeks. Body weight, food intake, and water intake were recorded weekly.

At the end of the experiment, fresh fecal samples were collected using sterile 1.5 mL microcentrifuge tubes and immediately stored at −80 °C for subsequent gut microbiota analysis. After behavioral assessments, the mice were anesthetized with tribromoethanol at a dose of 250 mg/kg body weight intraperitoneal injection. Blood samples were collected from the orbital plexus, and serum was separated by centrifugation. The mice were then perfused with saline, colon and hippocampal tissues were harvested and either fixed in 4% paraformaldehyde or stored at −80 °C for subsequent analyses. The study protocol was designed to minimize the number of animals used and their suffering and was approved by the Animal Care and Ethics Committee of Ningbo University (permission number: 12269).

### 2.4. SD Experimental Model

A chronic SD model was established using the multiple-platform water environment method [25]. The experimental apparatus consisted of a custom water tank (58 cm × 35 cm × 20 cm) uniformly fitted with multiple smooth cylindrical platforms (diameter 2.5 cm, height 8 cm) spaced 5 cm apart. Water was added to a depth of approximately 1 cm beneath the platforms, and the temperature was maintained at 23 ± 2 °C, with fresh water replaced daily. This setup allowed mice to move freely between platforms, but the platform surface was insufficient to support stable sleep. When mice entered the sleep state, muscle tone decreased, causing contact with the water surface and awakening, thereby achieving continuous sleep disruption through repeated arousal [26]. Mice in the SD, FLX, DO, and FDO groups underwent SD from 18:00 to 10:00 the following day (16 h per day) for 4 consecutive weeks. Mice in the NC group were maintained under the same environmental conditions without SD intervention. During the experimental period, all mice had free access to food and water, and their activity was monitored in real time to ensure animal safety.

### 2.5. Gut Microbiota Analysis

Total DNA from fresh fecal samples was extracted using the E.Z.N.A.^®^ Stool DNA Kit (Omega Bio-tek, Norcross, GA, USA) according to the manufacturer’s instructions [27]. The V3-V4 region of the 16S rRNA gene was amplified using primers 341F (5′-CCTACGGGNGGGCWGAG-3′) and 805R (5′-GACTACHVGGGTWTCTAATCC-3′). PCR conditions were as follows: initial denaturation at 98 °C for 30 s; 32 cycles of 98 °C for 10 s, 54 °C for 30 s, and 72 °C for 45 s; followed by a final extension at 72 °C for 10 min. PCR products were purified using AMPure XP magnetic beads (Beckman Coulter Genomics, Danvers, MA, USA) and quantified using a Qubit fluorometer (Invitrogen, Carlsbad, CA, USA). Qualified amplicons were subjected to paired-end 300 bp (PE300) sequencing on the DNBSEQ-G99 platform using the DNBSEQ-G99RS high-throughput sequencing kit (LC-Bio Technologies, Hangzhou, China). Raw sequencing data were processed by removing primer sequences, merging paired-end reads, filtering low-quality reads, and removing chimeras. Sequence denoising and generation of amplicon sequence variants (ASVs) were performed using DADA2. Taxonomic classification was conducted using the QIIME2 feature classifier against the SILVA database (version 138.2) and the NT-16S database. Alpha diversity, beta diversity, and taxonomic composition analyses were calculated, and relevant visualizations were performed using R software. Differential taxonomic features were further analyzed using STAMP and LEfSe, with an LDA score threshold of 3.0 used for LEfSe analysis.

### 2.6. Extraction and Analysis of SCFAs

Approximately 50 mg of fecal samples was weighed and mixed with an appropriate volume of 80% methanol. The samples were homogenized with steel beads, vortexed thoroughly, and centrifuged at 20,000 rpm for 5 min at 4 °C. Subsequently, 20 µL of the supernatant was transferred into a 1.5 mL centrifuge tube, followed by the sequential addition of EDC solution [1-ethyl-3-(3-dimethylaminopropyl) carbodiimide] and 3-NPH solution (3-nitrophenylhydrazine) for derivatization [28]. After derivatization, the mixture was brought to a final volume of 1 mL with the initial mobile phase solution, vortexed thoroughly, and 200 µL of the solution was transferred into an injection vial for liquid chromatography–tandem mass spectrometry (LC-MS/MS) analysis. Chromatographic separation was performed on a Poroshell 120 EC-C18 column (2.7 μm, 2.1 × 100 mm). The injection volume was 2 µL, the flow rate was 0.4 mL/min, and the column temperature was maintained at 40 °C. The mobile phase consisted of water as phase A and methanol:acetonitrile = 1:1 (*v*/*v*) as phase B, with gradient elution applied. Mass spectrometric data were acquired in electrospray ionization negative-ion mode, and quantitative analysis was performed using multiple reaction monitoring (MRM) in negative-ion mode.

### 2.7. Histological Examination

Fresh colon tissues were fixed overnight in 4% paraformaldehyde at 4 °C, dehydrated through a graded ethanol series, embedded in paraffin, and sectioned at a thickness of 3 μm. After deparaffinization with xylene, the sections were rehydrated sequentially with graded ethanol solutions at different concentrations ranging from 70% to 95% and finally washed with PBS (pH 7.4). Hematoxylin and eosin (H&E) staining was performed [29]. After mounting, the sections were observed and imaged under a light microscope. Histological injury was scored based on epithelial damage, crypt architecture, goblet cell loss, and inflammatory cell infiltration, with a total score ranging from 0 to 8.

### 2.8. Immunohistochemical and Immunofluorescence Analyses

For antigen retrieval, paraffin-embedded tissue sections with a thickness of 4 μm were baked at 65 °C for 1 h, deparaffinized twice in xylene for 10 min each, rehydrated through a graded ethanol series, and washed with PBS. The sections were placed in EDTA buffer (pH 9.0) for microwave-mediated antigen retrieval. After natural cooling, the sections were washed with PBS. Endogenous peroxidase activity was blocked with 3% hydrogen peroxide for 10 min, followed by washing and serum blocking for 30 min. After removal of the blocking solution, the sections were incubated with primary antibodies overnight at 4 °C. The following primary antibodies were used for immunohistochemistry: anti-ZO-1 (Proteintech, Rosemont, IL, USA 1:200, rabbit), anti-Occludin (Proteintech, 1:200, rabbit), anti-5-HT_1A_ receptor (HUABIO, Woburn, MA, USA, 1:200, rabbit), and anti-5-HT_2A_ receptor (HUABIO, 1:200, rabbit). For immunofluorescence, the primary antibodies were: anti-Iba1 (Wako, Richmond, VA, USA, 1:500, rabbit) and anti-GFAP (Cell Signaling Technology, Danvers, MA, USA, 1:400, mouse).

For immunohistochemical staining, after removal of the primary antibodies and washing, the sections were incubated with HRP-conjugated secondary antibodies for 50 min at room temperature in the dark. Color development was performed using a 3,3′-diaminobenzidine (DAB) chromogenic kit (Servicebio, Wuhan, China), followed by counterstaining, dehydration, clearing, and mounting. ImageJ software (version 1.53q, NIH, Bethesda, MD, USA) was used to calculate the average optical density (AOD) for quantitative assessment of ZO-1 and occludin expression in colon tissues and 5-HT_1A_ and 5-HT_2A_ expression in the hippocampus. For each sample, three high-power fields (HPFs) were randomly selected for analysis, and the mean value was used for statistical comparison. For immunofluorescence analysis, after washing with PBS, the sections were incubated with fluorescently labeled secondary antibodies for 50 min at room temperature in the dark. After washing with PBS, the sections were stained with 4′,6-diamidino-2-phenylindole (DAPI) for 10 min to label nuclei. The sections were then mounted with an anti-fluorescence quenching mounting medium and imaged under a fluorescence microscope [30]. ImageJ software was used to calculate the fluorescence intensity of glial cells in each group, and the values were normalized to those of the control group.

### 2.9. Western Blot Analysis

A total of 20 mg of hippocampal tissue was added to 100 μL of ice-cold lysis buffer containing 50 mM Tris–HCl (pH 7.4), 150 mM NaCl, 1 mM EDTA-2Na, 1% Triton X-100, 1% sodium deoxycholate, and 0.1% SDS, and homogenized by ultrasonication on ice. The lysate was centrifuged at 12,000 rpm for 5 min at 4 °C, and the supernatant was collected. Total protein concentration was determined using a BCA protein assay kit (Beyotime, Shanghai, China). Equal amounts of protein samples were separated by 10% SDS-PAGE and then transferred onto PVDF membranes using the wet transfer method. The membranes were blocked with 5% BSA for 2 h [31]. Subsequently, the membranes were incubated overnight at 4 °C with primary antibodies against PSD-95 (Proteintech, 1:10,000, rabbit), SYN (Proteintech, 1:40,000, rabbit), TPH_2_ (ABclonal, Woburn, MA, USA, 1:1000, rabbit), 5-HT_1A_ (HUABIO, 1:2000, rabbit), 5-HT_2A_ (HUABIO, 1:2000, rabbit), and β-actin (ZENBIO, Durham, NC, USA, 1:10,000, rabbit), which were diluted in blocking solution. The following day, the membranes were incubated with HRP-conjugated goat anti-rabbit secondary antibody (Biosharp, Beijing, China, 1:40,000) for 2 h at room temperature. After washing with TBST, the chemiluminescent substrate was added, and the signals were visualized using a gel imaging system. Protein bands were semi-quantitatively analyzed using ImageJ software, and β-actin was used as the internal reference for normalization.

### 2.10. RT-qPCR Analysis

Total RNA was extracted from frozen mouse colon tissues using an RNA extraction kit [32]. Briefly, 10–20 mg of colon tissue was homogenized in 500 μL of Buffer RL1 (FOREGENE, Cat. No. RE-03014). Genomic DNA was removed by centrifugation through a DNA-Cleaning Column, and RNA was purified using an RNA-Only Column and eluted with 50–200 μL of RNase-free ddH_2_O. RNA concentration and purity were measured using a NanoDrop 2000 spectrophotometer (Thermo Fisher Scientific, Waltham, MA, USA), and samples with A260/A280 ratios of 1.8–2.0 were used for reverse transcription. A total of 1 μg of total RNA was reverse-transcribed into cDNA using the All-in-One 5× RT MasterMix reverse transcription kit (ABM, New York, NY, USA, Cat. No. G592) under the following reaction conditions: 37 °C for 15 min, 60 °C for 10 min, and 95 °C for 3 min. qPCR was performed using a 2× qPCR Mix containing SYBR Green on a BIOER FQD-96C system. The 20 μL reaction system consisted of 10.0 μL of 2× qPCR Mix, 0.5 μL each of forward and reverse primers, 2.0 μL of cDNA, and 7.0 μL of DEPC-treated H_2_O. The amplification program was as follows: initial denaturation at 95 °C for 3 min; followed by 40 cycles of denaturation at 95 °C for 15 s and annealing at 60 °C for 20 s; and melting curve analysis from 60 °C to 95 °C, with an increment of 0.3 °C every 15 s. β-actin was used as the reference gene. For each sample, technical replicates were first averaged to obtain the mean Ct value. ΔCt was then calculated as Ct(target gene)—Ct(β-actin). The NC sample was used as the calibrator, and its mean ΔCt value was set as the baseline for ΔΔCt calculation. Relative mRNA expression levels of ZO-1 and Occludin were calculated using the 2^−ΔΔCt^ method.

### 2.11. Measurement of Biochemical Indicators

An appropriate amount of serum was collected, and hippocampal tissues were homogenized with pre-cooled homogenization buffer and centrifuged to obtain the supernatant. The levels of inflammatory factors, including IL-6, IL-1β, TNF-α, and IL-10, in serum and hippocampal tissues were detected according to the instructions of the ELISA kits. In addition, serum LPS and hippocampal BDNF, 5-HT, 5-HIAA, and CORT levels were also quantified using commercial ELISA kits.

### 2.12. Behavioral Assessment

Three behavioral tests were performed to evaluate spontaneous locomotor activity, anxiety-like behavior, and depressive-like behavior in mice.

#### 2.12.1. Open Field Test (OFT)

The open field test (OFT) was used to assess spontaneous locomotor activity and anxiety-like behavior in mice [33]. The apparatus consisted of an open-field box measuring 50 cm × 50 cm × 40 cm, with an infrared camera and a fan installed at the top for movement tracking and ventilation. During the test, each mouse was individually placed in the center of the arena, and its movement trajectory, total distance traveled, number of center crossings, and time spent in the center were recorded for 6 min. The experimental environment was kept quiet and dimly lit, with the temperature maintained at 23 ± 1 °C. After each test, the bottom of the box was cleaned with 75% ethanol to remove feces and odor cues and to prevent interference with subsequent tests.

#### 2.12.2. Elevated Plus Maze (EPM)

The elevated plus maze (EPM) was used to evaluate anxiety-like behavior in mice [34]. The apparatus consisted of two open arms (40 cm × 10 cm), two closed arms (40 cm × 10 cm × 30 cm), and a central platform. At the beginning of the test, each mouse was gently placed in the central area of the maze with its head facing an open arm. After release, the time spent in the open and closed arms was recorded. The percentage of time spent in the open arms was calculated as follows: open-arm time/(open-arm time + closed-arm time) × 100%. A decrease in the percentage of time spent in the open arms indicated enhanced anxiety-like behavior. After each mouse was tested, the maze was sprayed and cleaned with 75% ethanol to remove excreta.

#### 2.12.3. Tail Suspension Test (TST)

The tail suspension test (TST) was performed according to a standardized procedure to evaluate depressive-like behavior in mice. During the test, the tail of each mouse was fixed with paper tape and suspended approximately 35 cm above the tabletop. The tape was attached 2–3 cm from the tip of the tail, and the test lasted for 5 min. The total immobility time of each mouse was recorded in seconds. Immobility was defined as the absence of active struggling, with only passive hanging or slight swinging observed. A shorter immobility time is generally interpreted as an antidepressant-like effect.

### 2.13. Statistical Analysis

Data are presented as the mean ± standard deviation (SD). After confirming normality and homogeneity of variance, intergroup comparisons were performed using one-way analysis of variance (one-way ANOVA), followed by Tukey’s post hoc test for multiple comparisons (* *p* < 0.05, ** *p* < 0.01, *** *p* < 0.001, **** *p* < 0.0001). Statistical analyses were conducted using GraphPad Prism version 10.1.2 (GraphPad Software, San Diego, CA, USA).

## 3. Results

### 3.1. Chemical Composition Changes of Dendrobium officinale Juice After Fermentation

To elucidate the chemical basis for the altered bioactivity of FDO, the contents of total sugar, total acid, total phenolics, and GABA were compared between DO and FDO. As shown in Table 1, after mixed fermentation, the total sugar content decreased from 4.61 to 3.77 g/L, indicating carbohydrate consumption and possible utilization of polysaccharide-derived substrates during fermentation. Notably, total acid increased by approximately 4.6-fold, total phenolics nearly doubled, and GABA increased to 2.962 after fermentation. These results indicate that mixed fermentation reshaped the chemical profile of *Dendrobium officinale*, characterized by reduced sugar content and the accumulation of organic acids, phenolic compounds, and bioactive small molecules.

### 3.2. FDO Alleviated SD-Induced Body Weight Loss and Reduced Food Intake

**Figure 1:**
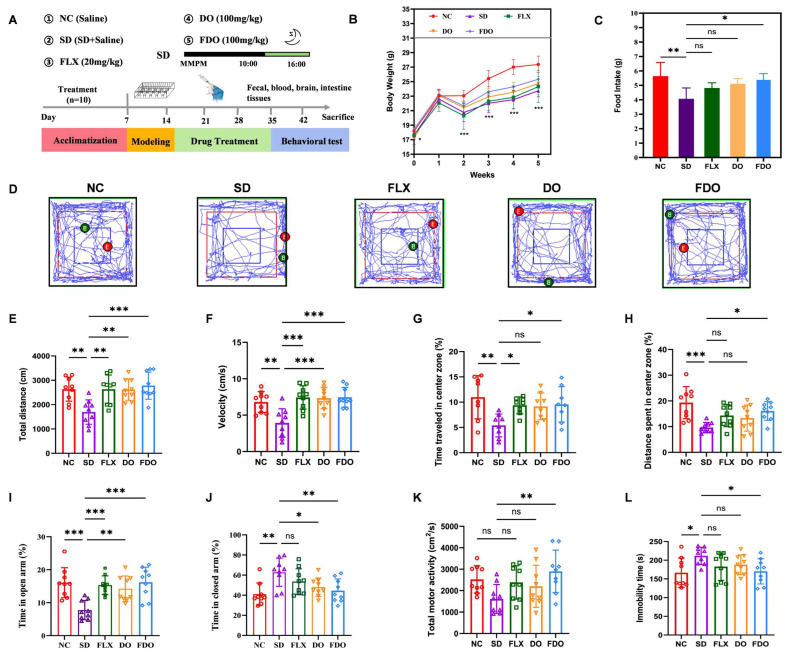
Physiological traits and behavioral performance in mice treated with FDO. (**A**) Experimental design. (**B**) Body weight of mice during the experimental period. (**C**) Average daily food intake. (**D**) Representative trajectories in the open-field test (OFT). (**E**) Total distance traveled in OFT. (**F**) Average velocity in OFT. (**G**) Percentage of the distance traveled in the central area relative to the total distance. (**H**) Percentage of the time spent in the central area relative to the total time. (**I**) Time spent in open arms. (**J**) Time spent in closed arms. (**K**) Total motor activity in the tail suspension test (TST). (**L**) Immobility duration in TST. Data are expressed as mean ± SD (*n* = 8–10). * *p* < 0.05, ** *p* < 0.01, *** *p* < 0.001, ns: no significant difference compared with the SD group. NC: normal control; SD: sleep deprivation; DO: unfermented *Dendrobium officinale*; FDO: fermented *Dendrobium officinale.*

During the 5-week period of standard chow feeding, body weight and food intake showed distinct trends among the groups. Compared with the NC group, mice in the SD group exhibited a marked decrease in body weight from week 2 onward and remained at a relatively low level throughout the experiment, suggesting that SD inhibited normal body weight gain in mice (Figure 1B). Both DO and FDO interventions alleviated SD-induced body weight loss to some extent, with a more pronounced recovery trend observed in the FDO group, whose overall body weight was higher than that of the DO group. Meanwhile, food intake was significantly reduced in the SD group compared with the NC group (*p* < 0.01) (Figure 1C). DO intervention slightly increased food consumption, but the difference was not statistically significant. In contrast, FDO significantly increased food intake (*p* < 0.05). Changes in body weight and food intake are general physiological indicators reflecting the overall health status of the organism [35]. These results suggest that SD impaired normal body weight gain and food intake, whereas FDO partially alleviated these physiological alterations.

### 3.3. FDO Alleviated SD-Induced Behavioral Abnormalities

To exclude the potential interference of motor dysfunction or other nonspecific behavioral alterations in the assessment of depressive-like behaviors, the OFT was first used to evaluate spontaneous locomotor activity and exploratory behavior in mice. As shown in Figure 1D, compared with the SD group, mice in the NC, FLX, DO, and FDO groups showed a greater tendency to explore the central area. Statistical analysis showed that the total distance traveled was significantly reduced in the SD group (*p* < 0.01), indicating impaired spontaneous locomotor activity [36]. In addition, the proportions of distance traveled and time spent in the central area were both significantly decreased in the SD group (*p* < 0.001 and *p* < 0.01, respectively), and the mean velocity was also markedly reduced (*p* < 0.01), suggesting that SD impaired spontaneous locomotor activity and induced anxiety-like behavior (Figure 1E–H). After supplementation with FDO and DO, all of these parameters were significantly improved, with a more pronounced improvement observed in the FDO group.

The EPM and TST further demonstrated the emotion-protective effects of the interventions from multiple behavioral dimensions. In the EPM test, SD mice spent significantly less time in the open arms (*p* < 0.001) and correspondingly more time in the closed arms (*p* < 0.01), further confirming that SD induced a pronounced anxiety-like phenotype (Figure 1I,J). In the TST, mice in the SD group also exhibited reduced overall activity and a significant prolongation of immobility time (*p* < 0.05). FDO intervention markedly reversed this behavioral despair-like phenotype and significantly shortened the immobility time of mice (*p* < 0.05) (Figure 1K,L). These findings indicate that SD successfully induced typical emotional abnormalities, including reduced spontaneous activity, anxiety-like avoidance, and behavioral despair, which are consistent with the core symptoms observed in patients with clinical depression. These results support the feasibility of the chronic SD-induced mouse model as a relevant animal model for depression research [37].

### 3.4. FDO Reshaped SD-Induced Gut Microbiota Dysbiosis

**Figure 2:**
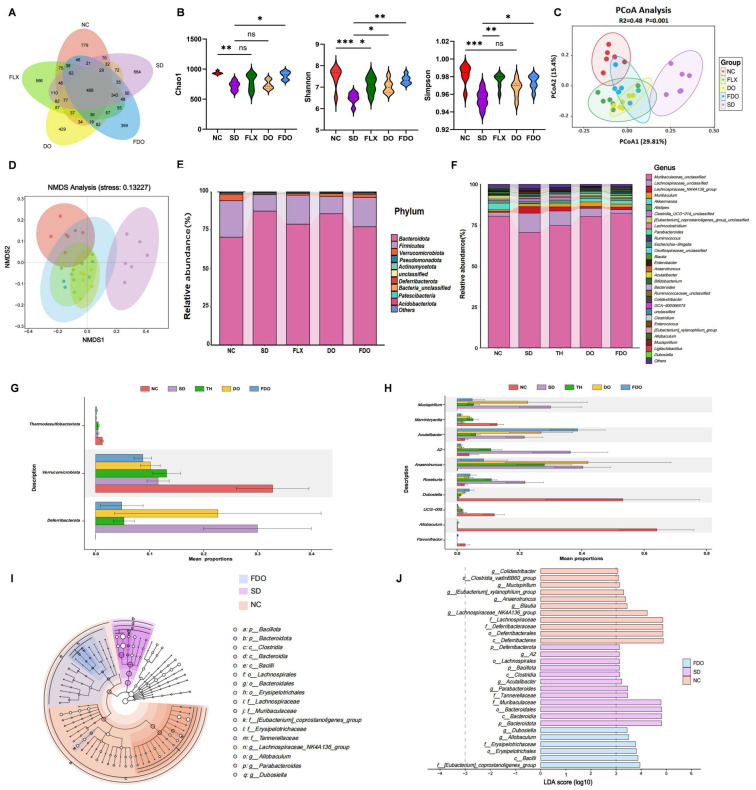
Effect of FDO supplementation on the gut microbiota in SD mice. (**A**) Venn diagram of ASVs for five groups. (**B**) α-diversity of Chao1, Shannon and Simpson. β−diversity analysis by calculating PCoA (**C**) and NMDS (**D**) analysis based on weighted UniFrac distances. The relative abundance of gut microbiota at the phylum (**E**) and genus (**F**) level in each group. (**G**) Differential abundance analysis of dominant phyla among five groups. (**H**) Differential genus-level analysis among five groups. (**I**) LEfSe identifying bacteria representative of differences among different groups. (**J**) Distribution histogram of LEfSe difference analysis (LDA > 3). Data are expressed as mean ± SD (*n* = 6). * *p* < 0.05, ** *p* < 0.01, and *** *p* < 0.001. ns: no significant difference compared with the SD group. NC: normal control; SD: sleep deprivation; DO: unfermented *Dendrobium officinale*; FDO: fermented *Dendrobium officinale.*

Previous studies have shown that dietary interventions, such as increased intake of dietary fiber, probiotics, and prebiotics, can improve the composition of the gut microbiota [38,39]. To investigate whether the alleviation of depressive-like behaviors by FDO was attributable to improvement of the gut microbial ecosystem, 16S rRNA gene sequencing was performed on fecal samples from mice in each group. Venn diagram analysis showed that the five groups shared 468 ASVs, whereas the NC, SD, FLX, DO, and FDO groups contained 779, 664, 566, 429, and 399 group-specific ASVs, respectively (Figure 2A). Alpha diversity analysis further showed that, compared with the NC group, the Chao1, Shannon, and Simpson indices were significantly decreased in the SD group (*p* < 0.01, *p* < 0.001, and *p* < 0.001, respectively), suggesting that SD reduced the richness and diversity of the gut microbiota (Figure 2B). After DO and FDO intervention, these indices recovered to varying degrees, with the FDO group being closer to the NC group. Principal coordinate analysis (PCoA) based on Bray–Curtis distance showed that samples within each group were relatively clustered, whereas the SD group was clearly separated from the NC group, indicating differences in microbial community structure among groups (Figure 2C). Similar results were obtained from NMDS analysis (Figure 2D). Both DO and FDO interventions partially reversed the SD-induced community shift, but did not completely restore the microbiota to the NC group state, suggesting that these interventions did not simply reconstruct the original microbial community but instead induced a specific microbial structure with distinct biological significance.

At the phylum level, *Bacteroidota*, *Firmicutes*, *Verrucomicrobiota*, *Pseudomonadota*, and *Actinomycetota* were dominant (Figure 2E). Notably, the SD group exhibited a decrease in *Firmicutes* abundance and an increase in *Bacteroidota* abundance, resulting in a reduced *Firmicutes* to *Bacteroidota ratio* (F/B). This alteration indicated that SD altered the composition of the gut microbiota [40]. Combined with the reductions in α-diversity, the separation of microbial communities in β-diversity analysis, these findings suggest that SD disrupted gut microbial homeostasis. After intervention with FDO or DO, the abundance of *Bacteroidota* decreased, whereas *Firmicutes* and *Verrucomicrobiota* showed an increasing trend, suggesting partial restoration of the SD-induced microbial community alteration.

At the genus level, *Muribaculaceae_unclassified*, *Lachnospiraceae_unclassified*, *Muribaculum*, and *Akkermansia* were the dominant taxa (Figure 2F). In the SD group, the abundance of *Muribaculaceae* was significantly decreased, whereas inflammation-associated genera such as *Alistipes* and *Lachnoclostridium* were increased, suggesting that SD-induced microbial dysbiosis may be accompanied by inflammatory activation [41]. *Muribaculaceae_unclassified* and *Akkermansia* showed increasing trends after DO and FDO intervention, suggesting partial remodeling of the microbial structure. In addition, STAMP analysis further confirmed differences in microbial composition among the groups, indicating that both DO and FDO exerted gut microbiota-modulating effects (Figure 2G,H).

To further identify characteristic microbial taxa associated with FDO intervention, LEfSe analysis was performed. The cladogram and LDA results showed that different groups had distinct dominant bacterial species and taxonomic units (Figure 2I,J). The SD group was mainly enriched in taxa such as *Parabacteroides* and *Bacteroidales*, whereas the FDO group was enriched in taxa closely related to intestinal metabolic function, including *Dubosiella*, *Allobaculum*, *Bacilli*, and *[Eubacterium]_coprostanoligenes_group.* These results indicate that FDO can reshape SD-induced gut microbiota dysbiosis. Its effect was reflected not only by a shift toward the microbial structure of the NC group, but also by the enrichment of specific functional bacterial taxa.

### 3.5. FDO Restored SCFAs Metabolic Homeostasis in SD Mice

**Figure 3:**
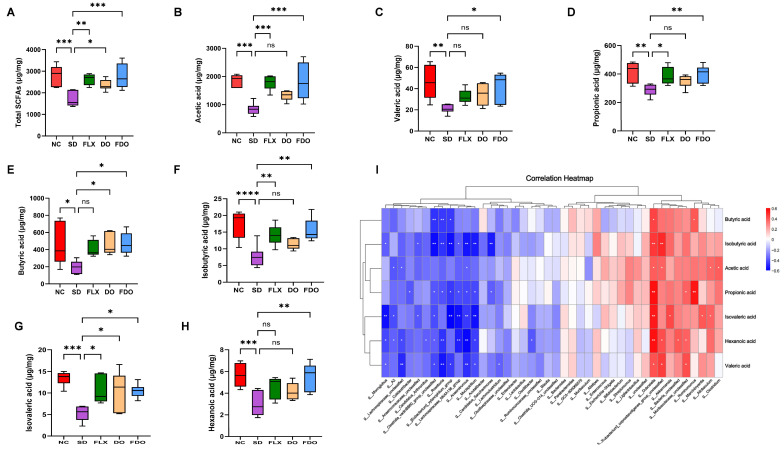
Effects of FDO on gut SCFA content in mice. (**A**–**H**) The levels of total short-chain fatty acids, acetic acid, valeric acid, propionic acid, butyric acid, isobutyric acid, isovaleric acid and hexanoic acid of the mouse fecal samples. (**I**) Heatmap of correlation analysis between SCFAs and gut microbiota. Data are expressed as mean ± SD (*n* = 6). * *p* < 0.05, ** *p* < 0.01, *** *p* < 0.001 and **** *p* < 0.0001. ns: no significant difference compared with the SD group. NC: normal control; SD: sleep deprivation; DO: unfermented *Dendrobium officinale*; FDO: fermented *Dendrobium officinale.*

To evaluate the relationship between gut microbiota and intestinal metabolic function, SCFA levels in mouse fecal samples were measured. As shown in Figure 3A–H, compared with the NC group, the total SCFA level in the feces of SD mice was markedly decreased (*p* < 0.001). Specifically, the levels of acetic acid (*p* < 0.001), valeric acid (*p* < 0.01), propionic acid (*p* < 0.01), butyric acid (*p* < 0.05), isobutyric acid (*p* < 0.0001), isovaleric acid (*p* < 0.001), and hexanoic acid (*p* < 0.001) were all significantly reduced, indicating that SD weakened the metabolic activity of the gut microbiota. Notably, FDO showed a more pronounced recovery trend in total SCFAs, acetic acid, and butyric acid levels than DO, suggesting that fermentation may enhance the regulatory effect of *Dendrobium officinale* on SCFA-related metabolic profiles in SD mice [42].

The correlations between SCFAs and the gut microbiota were further analyzed. The correlation heatmap showed that SCFA levels were significantly positively correlated with the relative abundances of bacterial taxa such as *Dubosiella*, *Akkermansia*, and *Ruminococcus* (Figure 3I). Combined with the 16S rRNA sequencing results, these findings suggest that FDO reversed SD-induced microecological metabolic imbalance by specifically enriching these key acid-producing genera. In addition, the gut microbiota can jointly maintain SCFA production through nutrient cross-feeding interactions [43]. For example, acetic acid produced by *Dubosiella* and *Akkermansia* during metabolism may serve as a substrate for butyrate-producing bacteria, thereby indirectly promoting butyric acid synthesis. These results indicate that both FDO and DO improved SD-induced abnormalities in the gut microbial ecosystem and metabolic function, with FDO exerting a more pronounced overall regulatory effect.

### 3.6. FDO Ameliorated SD-Induced Colonic Barrier Injury and Suppressed Peripheral Inflammation

**Figure 4:**
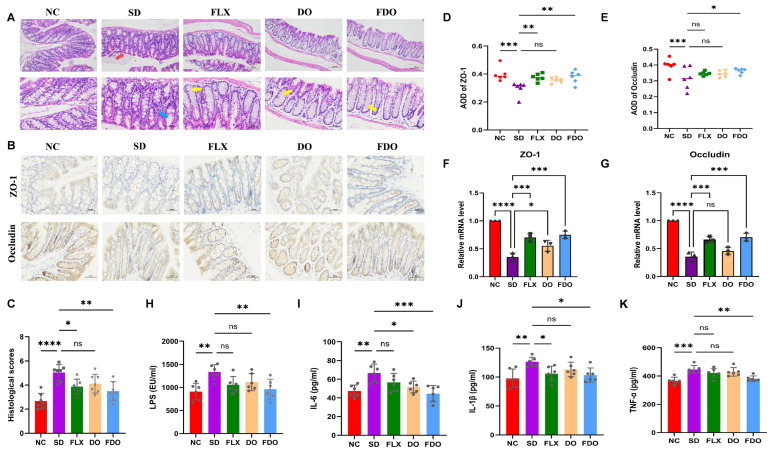
FDO ameliorates colon tissue damage and inflammatory responses. (**A**) H&E staining of the colon tissues. Scale bar = 100 µm and 50 µm. (**B**) Immunohistochemistry of ZO-1 and Occludin. Scale bar = 50 µm. (**C**) Histological scores of intestinal damage across groups. (**D**,**E**) Average optical density (AOD) of ZO-1 and Occludin. (**F**,**G**) The mRNA expression level of ZO-1 and Occludin. (**H**) Serum LPS levels. (**I**) Serum IL-6 levels. (**J**) Serum IL-1β levels. (**K**) Serum TNF-α levels. Data are expressed as mean ± SD (*n* = 3–6). The grey dots indicate the values of each independent experiment. * *p* < 0.05, ** *p* < 0.01, *** *p* < 0.001, and **** *p* < 0.0001. ns: no significant difference compared with the SD group. NC: normal control; SD: sleep deprivation; DO: unfermented *Dendrobium officinale*; FDO: fermented *Dendrobium officinale*.

The integrity of tissue morphology is fundamental for maintaining the physical barrier function of the intestine. Histopathological evaluation of mouse colon tissues by H&E staining showed that the colonic mucosa in the NC group was structurally intact, with regularly arranged glands (Figure 4A). In contrast, severe inflammatory lesions were observed in the colonic tissues of the SD group, mainly characterized by exfoliation of mucosal epithelial cells, irregular crypt architecture (red arrows), loss of goblet cells (yellow arrows), and increased numbers of lymphocytes in the lamina propria (blue arrows). After DO intervention, these histological injuries were partially alleviated, although a certain degree of mucosal structural abnormality remained. Supplementation with FDO more markedly improved colonic morphology, as evidenced by relatively intact mucosal epithelium, regular crypt architecture, increased numbers of goblet cells, and reduced inflammatory cell infiltration. Histological scoring further showed that SD significantly increased colonic injury, whereas FDO intervention reduced the injury score (Figure 4C).

The intestinal barrier consists of epithelial cells, the mucus layer, and tight junction proteins, such as ZO-1 and Occludin, and plays a critical role in maintaining intestinal homeostasis and preventing harmful substances from entering the circulation [44]. When the barrier is impaired, LPS can exacerbate inflammatory responses and further damage the epithelial barrier, thereby increasing intestinal permeability [45]. Immunohistochemical staining showed that ZO-1 and Occludin exhibited continuous linear or honeycomb-like punctate staining patterns in normal colonic mucosal epithelial cells (Figure 4B). Compared with the NC group, positive staining for both proteins was markedly reduced in the SD group, and the AOD values were significantly decreased (Figure 4D,E), suggesting that SD disrupted the colonic tight junction structure. In addition, qPCR results further confirmed that the mRNA expression levels of ZO-1 and Occludin were significantly decreased in the SD group compared with the NC group (*p* < 0.0001 and *p* < 0.0001, respectively) (Figure 4F,G). These alterations were partially reversed after FDO treatment, suggesting that FDO may help maintain intestinal barrier-related tight junction protein expression.

Consistent with barrier injury, the serum LPS concentration was significantly higher in the SD group than in the NC group (*p* < 0.001), indicating that SD promoted the entry of gut-derived endotoxin into the peripheral circulation. FDO intervention significantly suppressed the increase in LPS levels (*p* < 0.05), suggesting a potential alleviation of SD-associated intestinal barrier disruption. The entry of LPS into the bloodstream is a key trigger of systemic inflammatory responses. Therefore, we further quantified the serum levels of the pro-inflammatory cytokines IL-6, IL-1β, and TNF-α (Figure 4H–K). Compared with the NC group, the levels of IL-6 (*p* < 0.01), IL-1β (*p* < 0.01), and TNF-α (*p* < 0.001) were significantly increased in the SD group, indicating activation of systemic inflammation. Notably, FDO treatment effectively inhibited the excessive production of these inflammatory cytokines, and the level of IL-6 was even lower than that in the NC group.

### 3.7. FDO Restored Homeostasis of the Central 5-HT System

**Figure 5:**
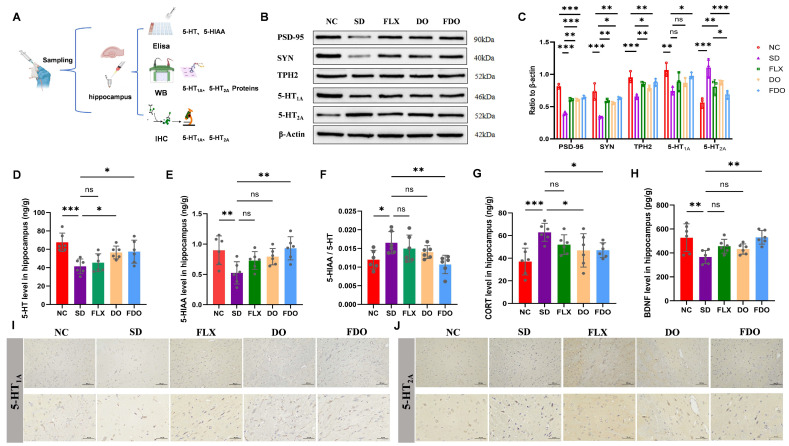
Effects of FDO on hippocampal 5-HT signaling, synaptic markers, and stress-related indicators in SD mice. (**A**) The experimental design. (**B**,**C**) Quantification of protein levels of PSD-95, SYN, TPH_2_, 5-HT_1A_ and 5-HT_2A_ in hippocampal tissue. (**D**) Hippocampal 5-HT levels. (**E**) Hippocampal 5-HIAA levels. (**F**) 5-HIAA/5-HT ratio. (**G**) Hippocampal CORT levels. (**H**) Hippocampal BDNF levels. The expressions of 5-HT_1A_ (**I**) and 5-HT_2A_ (**J**) in hippocampus tissue were stained with immunohistochemistry. Scale bar = 100 µm and 50 µm. Data are expressed as mean ± SD (*n* = 3–6). The grey dots indicate the values of each independent experiment. * *p* < 0.05, ** *p* < 0.01, and *** *p* < 0.001. ns: no significant difference compared with the SD group. NC: normal control; SD: sleep deprivation; DO: unfermented *Dendrobium officinale*; FDO: fermented *Dendrobium officinale*.

Peripheral inflammation can propagate to the central nervous system through the GBA and severely disrupt monoaminergic neurotransmitter homeostasis. Therefore, we further evaluated pathological alterations in 5-HT synthesis and metabolism, as well as its receptor network, in the central nervous system (Figure 5A). Western blot analysis showed that, after chronic SD stress, the hippocampal expression levels of 5-HT_1A_ and TPH_2_ were significantly decreased in mice (*p* < 0.01 and *p* < 0.001, respectively), whereas 5-HT_2A_ expression showed an abnormal pathological increase (*p* < 0.001), indicating that SD suppressed 5-HT synthetic capacity and caused dysregulation of receptor expression [46]. FDO treatment significantly ameliorated these alterations, restoring them to levels close to those in the NC group (Figure 5B,C).

The levels of 5-HT and its metabolite 5-HIAA in hippocampal tissues were further measured. Compared with the NC group, hippocampal levels of 5-HT and 5-HIAA were significantly decreased in the SD group (*p* < 0.001 and *p* < 0.01, respectively), whereas the 5-HIAA/5-HT ratio, which reflects neurotransmitter turnover and metabolic rate, was significantly increased (*p* < 0.05), suggesting that SD induced excessive degradation of 5-HT. Both FDO and DO groups showed a certain reversal trend, although the effect in the DO group did not reach statistical significance (Figure 5D–F). In addition, immunohistochemical staining further confirmed that 5-HT_1A_-positive signals were weakened and 5-HT_2A_-positive signals were enhanced in the hippocampus of the SD group, whereas FDO treatment corrected the imbalance between these two receptors (Figure 5I,J). These findings indicate that FDO can restore SD-induced abnormalities in hippocampal 5-HT synthesis, metabolism, and receptor expression, thereby improving homeostasis of 5-HT neurotransmission.

### 3.8. FDO Restored Hippocampal Synaptic Plasticity

Neuronal synaptic plasticity is fundamental for maintaining normal emotional behavior. The 5-HT signaling pathway not only regulates emotion and stress responses but also profoundly affects synaptic structure and function through specific receptors [47]. Therefore, after confirming the ameliorative effects of FDO on the 5-HT system, we further examined the expression of the presynaptic protein SYN and the postsynaptic protein PSD-95 in the hippocampus and evaluated changes in BDNF and CORT-related indicators. Western blot analysis showed that, compared with the NC group, the protein expression levels of hippocampal SYN (*p* < 0.001) and PSD-95 (*p* < 0.001) were significantly decreased in the SD group (Figure 5B,C), suggesting that SD impaired hippocampal synaptic plasticity. FDO intervention significantly upregulated the expression of SYN and PSD-95 (*p* < 0.01 and *p* < 0.001, respectively). In addition, BDNF levels in the hippocampus were markedly reduced in SD mice (*p* < 0.01), accompanied by a significant increase in CORT levels (*p* < 0.05), indicating that SD induced activation of the hypothalamic–pituitary–adrenal (HPA) axis stress response and a deficiency in local neurotrophic support (Figure 5G,H). Notably, FDO intervention not only effectively suppressed excessive CORT accumulation but also significantly restored BDNF levels, promoted the reconstitution of pre- and postsynaptic membrane proteins, and thereby comprehensively reversed SD-induced impairment of hippocampal synaptic plasticity.

### 3.9. FDO Inhibited SD-Induced Glial Activation and Neuroinflammation

**Figure 6:**
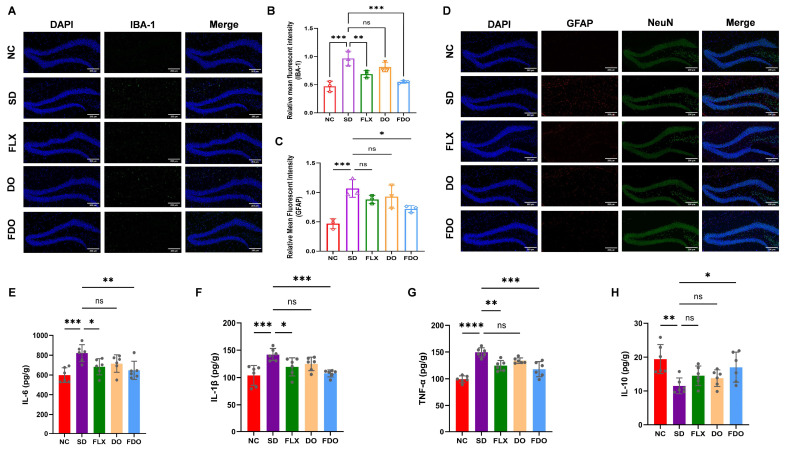
Effect of FDO on glial cell activation and inflammatory cytokine levels in SD mice. (**A**) Representative immunofluorescence images of IBA-1 (green, microglia) and DAPI (blue, nuclei) in the hippocampus.(**B**) Quantification of IBA-1 fluorescence intensity. (**C**) Quantification of GFAP fluorescence intensity. (**D**) Representative immunofluorescence images of GFAP (red, astrocyte), NeuN (green, neuronal nuclei), and DAPI (blue, nuclei) in the hippocampus. (**E**) Hippocampal IL-6 levels. (**F**) Hippocampal IL-1β levels. (**G**) Hippocampal TNF-α levels. (**H**) Hippocampal IL-10 levels. Data are expressed as mean ± SD (*n* = 3–6). Scale bar = 200 μm. The grey dots indicate the values of each independent experiment. * *p* < 0.05, ** *p* < 0.01, *** *p* < 0.001, and **** *p* < 0.0001. ns: no significant difference compared with the SD group. NC: normal control; SD: sleep deprivation; DO: unfermented *Dendrobium officinale*; FDO: fermented *Dendrobium officinale*.

Studies have shown that imbalance of the immune microenvironment in the central nervous system, particularly aberrant inflammatory activation of glial cells, is a core upstream mechanism driving dysregulation of neurotransmitter signaling and synaptic structural damage [48]. To evaluate whether SD impaired synaptic plasticity and aggravated depressive-like behaviors by inducing neuroinflammation, immunofluorescence staining was performed to detect the expression levels of Iba-1 and GFAP. As shown in Figure 6A,B, compared with the NC group, SD mice exhibited pronounced glial activation in the hippocampus, particularly in the CA1 and dentate gyrus (DG) regions. Quantitative analysis showed that the fluorescence intensity of Iba-1-positive microglia was increased in the SD group (*p* < 0.001), accompanied by a morphological transition from a ramified resting state to an activated-like phenotype characterized by enlarged cell bodies and reduced processes [49]. Meanwhile, the fluorescence intensity of GFAP-positive astrocytes was increased (*p* < 0.001), with marked reactive astrogliosis and enhanced astrocytic activation (Figure 6C,D) [50]. Compared with the SD group, FDO intervention significantly reduced the fluorescence intensities of Iba-1 and GFAP (*p* < 0.001 and *p* < 0.05, respectively).

Further analysis of hippocampal inflammatory cytokine levels showed that, compared with the NC group, the levels of TNF-α (*p* < 0.0001), IL-6 (*p* < 0.001), and IL-1β (*p* < 0.001) were significantly increased in SD mice, whereas the level of the anti-inflammatory cytokine IL-10 was decreased (*p* < 0.01), confirming that SD triggered central neuroinflammation (Figure 6E,H). FDO treatment significantly improved the expression profile of these inflammatory markers, with particularly pronounced inhibitory effects on IL-1β and TNF-α (*p* < 0.001). These results indicate that FDO can inhibit SD-induced excessive activation of hippocampal glial cells, reduce pro-inflammatory cytokine levels, and restore the expression of the anti-inflammatory factor IL-10, thereby alleviating central neuroinflammation.

### 3.10. Correlation Network Revealed the Overall Advantage of FDO in Regulating the GBA and the 5-HT System

**Figure 7:**
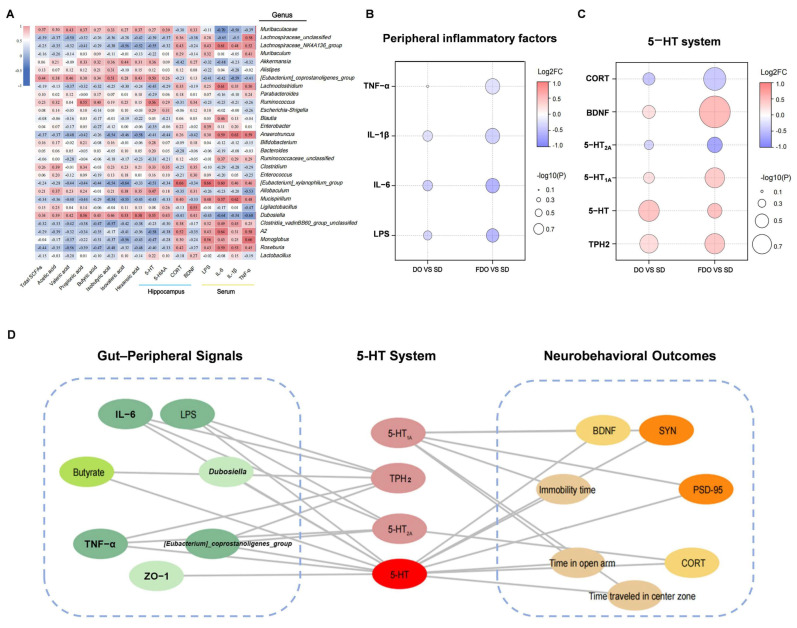
FDO fermentation enhancement and multi-indicator network analysis of 5-HT. (**A**) Spearman correlation analysis of gut microbiota with depression-related features. (**B**) Comparison of the effects of FDO and DO on peripheral inflammatory factors in SD mice. (**C**) Comparison of the regulatory effects of FDO and DO on the 5-HT system and related markers in SD mice. (**D**) Schematic diagram illustrating the GBA mechanism by which FDO alleviates SD-induced depression-like behavior. Data are expressed as mean ± SD (*n* = 6). DO: unfermented *Dendrobium officinale*; FDO: fermented *Dendrobium officinale.* Different colors denote different indicators, while the same color indicates items belonging to the same category.

To further elucidate the relationships between gut microbiota alterations and SD-induced depressive-like behavior-related indicators, Spearman correlation analysis was performed to construct a microbiota–metabolite–inflammation—5-HT association network. As shown in Figure 7A, *Muribaculaceae*, *Dubosiella*, and *Ruminococcus* were positively correlated with the levels of 5-HT, BDNF, and multiple SCFAs, but negatively correlated with LPS, IL-1β, TNF-α, and IL-6. *Akkermansia* was significantly positively correlated with acetic acid, isobutyric acid, 5-HT, and BDNF. In contrast, bacterial taxa such as *Lachnoclostridium*, *Anaerotruncus*, *Mucispirillum*, *A2*, and *Monoglobus* were significantly positively correlated with pro-inflammatory cytokines, including IL-1β, TNF-α, and IL-6, but negatively correlated with 5-HT, BDNF, 5-HIAA, and various SCFAs. Notably, the beneficial bacteria enriched in the FDO group, including *Dubosiella* and *[Eubacterium]_coprostanoligenes_group*, were significantly positively correlated with 5-HT, BDNF, and total SCFAs, and significantly negatively correlated with pro-inflammatory cytokines. Together with their higher abundance in the FDO group and the recovery of SCFA levels, these findings suggest that these bacterial taxa may be involved in FDO-mediated improvement of microbial metabolism, alleviation of inflammation, and restoration of hippocampal 5-HT and BDNF levels.

Based on the correlation analysis, bubble plots were generated for peripheral inflammation- and 5-HT system-related indicators to further compare the corrective effects of FDO and DO on SD-induced abnormalities. As shown in Figure 7B, compared with the SD group, both FDO and DO showed significant negative associations with inflammatory indicators, indicating that both interventions reduced inflammatory factor levels to some extent. However, FDO showed a greater corrective effect on indicators such as TNF-α and LPS, suggesting that fermentation may enhance the effect of DO in ameliorating gut-derived endotoxin translocation and peripheral inflammatory responses. Further analysis of 5-HT system-related indicators showed that FDO significantly upregulated the levels of 5-HT, TPH_2_, 5-HT_1A_, and BDNF, while downregulating 5-HT_2A_ and CORT (Figure 7C). In contrast, the overall effect of DO was weaker, with less pronounced statistical significance. These results indicate that FDO more comprehensively improved SD-induced abnormalities in microbial metabolism, inflammatory responses, and hippocampal 5-HT system dysregulation than DO, suggesting that fermentation may be an important factor enhancing the antidepressant-like effects of *Dendrobium officinale.*

## 4. Discussion

This study established a comprehensive mechanistic framework and systematically elucidated the specific role of FDO in targeting the GBA to suppress neuroinflammation and restore 5-HT synthesis, thereby alleviating chronic SD-induced depressive-like behaviors. Chronic SD has been identified as a major risk factor for the onset and exacerbation of depression [51,52]. In addition, differences in food intake among groups may have partly contributed to the observed changes in body weight, gut microbiota, and SCFA profiles. In the present study, SD mice also exhibited typical depressive-like behaviors, including reduced willingness to explore the central area in the OFT, shortened exploration time in the open arms of the EPM, and markedly prolonged immobility time in the TST. These parameters are well-established indicators of anxiety- and depression-related behaviors in rodents [53]. Notably, FDO intervention robustly reversed the above behavioral despair and anxiety-like phenotypes, with an overall effect superior to that of DO, suggesting that fermentation enhanced the protective effect of *Dendrobium officinale* against SD-related emotional and behavioral disturbances. Although FLX was used as a classical antidepressant positive control, its effects were parameter-dependent in the present study. Specifically, FLX significantly improved open-arm time in the EPM but did not significantly affect closed-arm time or TST parameters.

Reconstruction of gut microbial homeostasis is an important basis for the neuroprotective effects of FDO. Chronic SD commonly induces reduced gut microbial diversity and structural dysbiosis, further contributing to systemic metabolic disorders and persistent neuroinflammation [54]. In the present study, 16S rRNA sequencing showed that SD significantly reduced the diversity and richness of the gut microbiota and caused a marked shift in the overall community structure. After FDO intervention, both microbial diversity and community structure were restored to varying degrees, suggesting that the amelioration of SD-induced depressive-like behaviors may be closely associated with gut microbiota remodeling [55]. In addition, we found that FDO increased the relative abundances of *Firmicutes* and *Verrucomicrobiota* at the phylum level and reduced the abundance of inflammation-associated taxa such as *Deferribacterota*. Further analysis showed that FDO reversed the SD-induced decreases in *Muribaculaceae*, *Lachnospiraceae_NK4A136_group*, and *Muribaculum*, while suppressing the enrichment of inflammation-associated taxa such as *Alistipes* and *Parabacteroides. Muribaculum* is a major mucin monosaccharide-foraging bacterium that can inhibit pathogen colonization and exert anti-inflammatory effects, thereby helping to prevent metabolic disorders [56]. Meanwhile, *Muribaculaceae*, a bacterial family enriched in the healthy gut, has potential anti-inflammatory effects [57]. *Lachnospiraceae_NK4A136_group* is considered to be associated with SCFA production and intestinal barrier protection, and its restoration may contribute to the improvement of SD-induced microbial metabolic dysfunction. Notably, LEfSe analysis showed that FDO enriched characteristic taxa such as *Dubosiella* and *Allobaculum*. Previous studies have indicated that *Dubosiella* may be involved in the regulation of 5-HT synthesis and metabolism [58], whereas *Allobaculum* has also been shown to be significantly positively correlated with multiple microbial metabolites with neuroprotective effects, contributing to the improvement of neurotransmitter regulation [59]. In addition, although *Akkermansia* was not identified as a significantly differential taxon, its abundance tended to be higher in the FDO group than in the SD group. *Akkermansia* is closely associated with mucosal barrier repair, SCFA metabolism, and inflammation alleviation [60]. Therefore, FDO did not simply restore the gut microbial composition; rather, it enriched a series of functional taxa associated with SCFA production, barrier protection, and 5-HT regulation, thereby establishing a new beneficial microecological state. This may constitute an important basis for its superiority over DO.

Previous studies have suggested that SD disrupts microbial circadian rhythms, leading to reduced SCFA production and disturbed metabolic homeostasis [61]. Consistently, our study found that the fecal levels of total SCFAs, as well as acetic acid, propionic acid, butyric acid, isobutyric acid, valeric acid, isovaleric acid, and hexanoic acid, were significantly decreased in SD mice, suggesting that SD impaired the metabolic function of the gut microbiota. Among these metabolites, butyric acid and propionic acid have been widely recognized as being associated with host immune regulation and central nervous system modulation. Butyric acid can influence GBA function by regulating histone deacetylation, enhancing the expression of tight junction proteins, and suppressing inflammatory responses, whereas propionic acid has also been reported to participate in immune regulation and the control of neuroinflammation [62]. Therefore, the reduction in SCFAs may represent an important metabolic basis for SD-induced intestinal barrier injury, inflammatory activation, and emotional behavioral abnormalities. FDO intervention significantly restored fecal SCFA concentrations, reflecting improvement of gut microbial metabolism, which may be associated with the observed alleviation of intestinal barrier injury and neuroinflammation [63]. It should also be noted that FDO is a fermented product, and fermentation-derived organic acids, including lactic acid and other SCFA-related metabolites, may also contribute to the observed changes in SCFA levels. Correlation analysis further showed that SCFA levels were positively correlated with bacterial taxa such as *Dubosiella*, *Akkermansia*, and *Muribaculum.* Among them, *Dubosiella* may participate in SCFA production, whereas *Akkermansia* and *Muribaculum* are closely associated with mucus layer metabolism, intestinal barrier homeostasis, and SCFA-related metabolism, particularly showing positive correlations with isobutyric acid and valeric acid levels.

Mechanistically, SD may contribute to the development of depression by impairing neuroplasticity and inducing neuroinflammation [64]. SD not only weakens neural network connectivity and impairs memory consolidation and learning ability, but also increases the levels of pro-inflammatory cytokines, thereby affecting brain function and emotional regulation [65]. Pro-inflammatory cytokines can further cross the BBB, increase its permeability, and exacerbate neuroinflammation [66]. This inflammatory response mainly affects emotion-related brain regions, including the hippocampus, amygdala, and prefrontal cortex, and interferes with neurotransmitter metabolism [67]. Consistent with previous findings, we found that the levels of pro-inflammatory factors, including IL-1β, IL-6, and TNF-α, were significantly increased in both the serum and hippocampus of SD mice, suggesting that SD successfully induced peripheral inflammation and central neuroinflammation [68]. In addition, SD mice exhibited colonic tissue injury, decreased expression of ZO-1 and Occludin, and elevated serum LPS levels, indicating that SD disrupted intestinal barrier integrity and promoted the entry of gut-derived endotoxin into the bloodstream. FDO intervention restored the expression of tight junction proteins and reduced serum LPS levels as well as peripheral pro-inflammatory cytokine levels, suggesting that FDO may block the progression from SD-induced gut-derived inflammation to central inflammation through this pathway.

Once peripheral inflammatory factors breach the BBB, they can further exacerbate local neurotoxicity through excessive activation of central glial cells, representing a key pathological hub through which SD drives depressive-like behaviors. Immunofluorescence analysis showed that Iba-1 and GFAP-positive signals were markedly enhanced in the hippocampus of SD mice, accompanied by increased activation of microglia and astrocytes. Morphologically, glial cells shifted from a ramified resting phenotype toward an activated-like phenotype characterized by enlarged cell bodies and reduced processes. FDO intervention significantly reduced the expression of Iba-1 and GFAP and shifted glial cell morphology toward a resting-like state. Meanwhile, FDO downregulated the hippocampal levels of IL-1β, IL-6, and TNF-α, indicating that its neuroprotective effects partially depend on the inhibition of hippocampal glial activation and neuroinflammation.

In addition, monoaminergic neurotransmitters, particularly dopamine (DA), norepinephrine (NE), and 5-HT, play crucial roles in physiological functions. Among them, abnormalities in the 5-HT system have long been regarded as an important neurobiological basis for the development and progression of depression [69]. 5-HT function depends not only on the level of the neurotransmitter itself, but is also jointly regulated by synthetic enzymes, metabolic processes, and receptor signaling [70]. 5-HT_1A_ and 5-HT_2A_ are receptor subtypes closely associated with emotional regulation in the central nervous system, and they participate in the regulation of sleep and emotion by modulating 5-HT release [71]. In the present study, we focused primarily on the hippocampus and found that SD mice exhibited significantly reduced 5-HT levels and 5-HT_1A_ protein expression, whereas 5-HT_2A_ protein expression was significantly increased. These findings suggest that SD not only suppresses 5-HT synthetic capacity but also disrupts 5-HT metabolism and receptor expression homeostasis, which is consistent with previous reports [72]. We observed that FDO treatment effectively restored the levels of 5-HT, TPH_2_, and 5-HIAA and corrected the imbalance between 5-HT_1A_ and 5-HT_2A_ receptors in SD mice, suggesting that FDO ameliorates SD-induced dysregulation of the 5-HT system at multiple levels, reduces central excitability, and improves depressive-like behaviors, in agreement with earlier findings [73]. Notably, fluoxetine, as a selective serotonin reuptake inhibitor, exerts its antidepressant effects mainly by enhancing 5-HT signaling in the synaptic cleft. However, when severe neuroinflammation impairs 5-HT synthesis, simply blocking reuptake may have certain limitations [74]. In contrast, FDO may relieve the pathological inhibition of TPH_2_ by inflammatory factors through suppression of glial cell-driven neuroinflammation. Meanwhile, FDO improves 5-HTergic system homeostasis through multiple GBA-related targets, including modulation of the gut microbiota, restoration of SCFAs, and protection of the intestinal barrier. These findings suggest that FDO does not simply increase 5-HT levels, but rather promotes 5-HT synthesis and reconstruction of receptor signaling by suppressing the inflammatory microenvironment and restoring GBA homeostasis, thereby improving SD-induced depressive-like behaviors at multiple levels.

At the molecular level, synaptic plasticity optimizes the efficiency of neural signal transmission by regulating the strength of synaptic connections and represents an important component of emotional behavioral regulation [75]. SYN, as a presynaptic plasticity-associated protein, reflects synaptic transmission efficiency through its expression level. PSD-95 is an important postsynaptic scaffold protein that plays a key role in maintaining the structural stability of excitatory synapses and synaptic signal transduction [76]. In the present study, FDO treatment significantly restored the expression levels of both synaptic proteins, indicating that FDO may help recover the expression of synaptic plasticity-related molecules and thereby improve depressive-like behaviors. BDNF is an important neurotrophic factor involved in the regulation of neuronal survival, synaptic plasticity, and neurogenesis. By binding to its high-affinity receptor TrkB, BDNF can activate downstream signaling pathways, such as CREB, thereby promoting synaptic protein expression and neural network remodeling [77]. In this study, FDO significantly increased hippocampal BDNF levels. Notably, BDNF and the 5-HT system exhibit bidirectional regulatory interactions: on the one hand, BDNF provides neurotrophic support for 5-HTergic neurons; on the other hand, 5-HT can promote BDNF expression by activating 5-HT_1A_ receptor signaling [78]. In our results, FDO simultaneously restored the levels of 5-HT, 5-HT_1A_, and BDNF, suggesting that these pathways may form a positive synergistic interaction. In addition, both neuroinflammation and chronic stress can impair BDNF-related signaling and weaken synaptic plasticity [79]. The present study confirmed that FDO significantly inhibited the elevation of pro-inflammatory cytokines, including IL-6, IL-1β, and TNF-α, while reducing CORT levels. As an important marker of the HPA axis stress response, persistently elevated CORT can impair hippocampal neuronal survival and synaptic plasticity, whereas reducing CORT levels may help restore hippocampal structural and functional homeostasis [80]. Therefore, FDO may exert antidepressant-like effects by suppressing neuroinflammation, reducing CORT levels, restoring the 5-HT system, activating BDNF signaling, and enhancing synaptic plasticity.

Our study provides an integrated perspective and makes a novel contribution to understanding SD-induced depressive-like behaviors. Unlike previous studies that have focused on a single aspect of this complex system, we systematically investigated the interactions among the gut microbiota, SCFAs, intestinal barrier, peripheral and central neuroinflammation, 5-HT synthesis and metabolism, 5-HT_1A_/5-HT_2A_ receptor expression, BDNF, synaptic plasticity, and behavioral outcomes. Based on these findings, we constructed a schematic diagram of the intervention mechanism centered on 5-HT (Figure 7D), which visually summarizes the regulatory network extending from upstream gut-derived signals to downstream neurobehavioral outcomes. Notably, compared with DO, FDO exhibited more pronounced overall advantages in microbiota remodeling, SCFA restoration, inflammation suppression, and regulation of the 5-HT system. These advantages may be attributed to fermentation-induced transformation of active constituents and the enrichment of bacterial taxa such as *Dubosiella* and *Allobaculum*, which are associated with SCFA production, intestinal barrier repair, and neurotransmitter regulation, and may jointly contribute to positive modulation of the GBA [81].

The present study has several limitations. First, germ-free mouse colonization or fecal microbiota transplantation experiments were not performed to directly verify the causal relationship between gut microbiota alterations and behavioral phenotypes. In addition, although correlation analysis suggested potential associations among gut microbial taxa, SCFAs, inflammatory markers, and 5-HT-related indicators, these findings do not establish direct causal relationships and should be interpreted as exploratory associations. Second, the study focused on comparing the overall effects of DO and FDO; however, placebo controls using fermented culture medium without *Dendrobium officinale*, probiotic-only groups, and sleep recovery groups were not included. This limitation prevents fully distinguishing the specific contributions of fermentation medium, probiotic cells, and spontaneous recovery after SD. Third, FDO exhibited more pronounced intervention effects than DO, yet the specific fermentation-derived components responsible for these enhanced effects remain to be identified [82]. Fourth, the chronic SD model reproduced depression-like phenotypes, but it cannot fully reflect the complex etiology and clinical heterogeneity of human depression, so its construct validity should be interpreted with caution. Finally, the long-term effects and toxicological safety of FDO administration remain undetermined; future studies using humanized microbiota models and advanced multidimensional omics technologies, including single-cell approaches, will help more precisely elucidate the cell-specific regulatory network of FDO and facilitate its clinical translation.

## 5. Conclusions

In conclusion, FDO effectively alleviated SD-induced depressive-like behaviors, and its effects were closely associated with multilevel regulation of the GBA. FDO attenuated excessive inflammatory responses by reshaping the gut microbiota structure and its metabolites, particularly SCFAs, and further suppressed glial activation and neuroinflammatory responses. At the central nervous system level, FDO restored TPH_2_-mediated 5-HT synthesis, the homeostasis of 5-HT_1A_/5-HT_2A_ receptor expression, BDNF levels, and the expression of synaptic plasticity-related proteins, thereby improving SD-induced neurological dysfunction. Our findings indicate that the gut microbiota and the 5-HT system are key regulatory factors in SD-induced depressive-like behaviors and that FDO exhibits more pronounced overall advantages in systemic restoration. These findings suggest that FDO may serve as a promising nutritional candidate for preventing and ameliorating chronic SD-related depressive-like behaviors and provide experimental evidence for the development of GBA-targeted intervention strategies.

## Figures and Tables

**Table 1:** Comparison of total sugar, total acid, total phenolics, and GABA contents between DO and FDO.

	Total Sugar (g/L)	Total Acid (g/L)	Total Phenol (mg/L)	GABA (mg/mL)
DO	4.61 ± 0.04	2.55 ± 0.08	26.12 ± 1.43	0.328 ± 0.005
FDO	3.77 ± 0.06	11.74 ± 0.51	52.77 ± 0.41	2.962 ± 0.026

Data are expressed as mean ± SD (*n* = 3). DO: unfermented *Dendrobium officinale*; FDO: fermented *Dendrobium officinale.*

## Data Availability

The datasets generated for this study are available on request to the corresponding authors.
